# Postpartum women’s views on human milk banking in a city in Southeast China: a cross-sectional survey

**DOI:** 10.1186/s13006-021-00443-8

**Published:** 2022-01-07

**Authors:** Haixia Tu, Ping Li, Lianlian Zhu, Xiaozhen Quan, Shuli Fan, Ziyue Wang

**Affiliations:** 1 School of Nursing, Wenzhou Medical University, Wenzhou, Zhejiang China; 2 Department of Obstetrics, Second Affiliated Hospital of Wenzhou Medical University, Wenzhou, Zhejiang China; 3 Department of Obstetrics, First Affiliated Hospital of Wenzhou Medical University, Wenzhou, Zhejiang China

**Keywords:** Human milk bank, Donor human milk, Breastfeeding, Willingness, Knowledge, Factors, China

## Abstract

**Background:**

Donor human milk is the best alternative for preterm infants when their mother’s own milk is insufficient or unavailable. The development of human milk banks in China started late, and in most of these banks, the amount of donor human milk is insufficient for clinical demand. Moreover, many mothers are reluctant to use donor human milk due to safety concerns. It is important to understand the potential supply and demand of donor human milk before establishing a new human milk bank. This study aimed to understand women’s acceptance of human milk banking in Wenzhou, southeastern China.

**Methods:**

A cross-sectional study was conducted in three community health centers in Wenzhou, southeast China, in December 2020. Data were collected from 305 postpartum women selected through convenience sampling. Sociodemographic, perinatal and breastfeeding characteristics, awareness and knowledge of human milk banking and willingness to donate human milk, and to accept donor human milk were assessed. Multivariable logistic regression analysis was used to explore independent predictors of willingness to donate human milk and to accept donor human milk.

**Results:**

Only 17% (52/305) of our participants had heard of human milk banking prior to this survey. The prevalence of willingness to donate human milk and use donor human milk in our study was 73.4% (224/305) and 44.6% (136/305), respectively. Employment (adjusted odds ratio [AOR] 2.30; 95% confidence interval [CI] 1.17, 4.50) and human milk banking knowledge (AOR 1.23; 95% CI 1.12, 1.35) were independent predictors of willingness to donate human milk. Monthly household income in the previous year (AOR 2.18; 95% CI 1.17, 4.06), awareness of human milk banking (AOR 2.41; 95% CI 1.24, 4.67) and knowledge of human milk banking (AOR 1.22; 95% CI 1.11, 1.35) were significantly associated with willingness to accept donor human milk.

**Conclusions:**

In our study, awareness of human milk banks among women in the first year postpartum was low. More mothers were willing to donate human milk than to use donor human milk to feed their children. In our study, knowledge of human milk banking was a predictor of both willingness to donate human milk and willingness to use donor human milk. Programs with detailed information on human milk banking are needed to help mothers improve their knowledge and increase acceptance of human milk banking.

## Background

Since the implementation of China’s new fertility policy [1], which advocates for two children per couple, the number of older pregnant women has increased, as has the number of preterm infants [2]. Due to the rapid development of technology in perinatology and neonatology, an increasing number of preterm infants, especially those with very low birthweight, are successfully treated in the neonatal intensive care unit (NICU). Nutritional support plays an important role in the successful treatment of preterm infants, and breastfeeding is the best option for preterm infants [3]. However, the relatively long hospital stay for premature infants and the traditional Chinese practice of “confinement” after birth, which requires the mother to recuperate at home or in a maternity hotel, result in the separation of the mother and infant for a long time after birth [4, 5]. The separation leads to the mother’s breasts not being stimulated by suckling, reducing the secretion of prolactin and leading to inadequate breast milk production [6]. As a result, many premature infants in China do not receive their mothers’ own milk during their stay in the hospital [7, 8]. Some studies have been conducted to help women breastfeed preterm infants with their own milk and have achieved positive results [9–11]. However, there are still cases where mothers are unable to breastfeed their infants.

Donor human milk (DHM) from a formal human milk bank (HMB) is recommended by professional health organizations as a preferred substitute when a mother’s own milk is insufficient or unavailable [12–15]. Studies have shown that DHM can reduce the incidence of necrotizing enterocolitis and improve feeding tolerance [16–19]. The use of DHM for preterm and very low birthweight infants is increasing worldwide [16, 20–24]. The creation of HMBs is important in promoting breastfeeding in the NICU and fully embodies the goals of the Baby-Friendly Hospital Initiative.

The development of HMBs in China started late, with the first HMB established in Guangzhou in March 2013 [25], followed by a rapid development phase, leading to the development of a total of 19 HMBs by October 2018 [26]. However, most HMBs in China are faced with the dilemma that the number of milk donors and the amount of DHM are far from meeting clinical demand [27]. Moreover, many mothers in China are reluctant to use DHM to feed their children due to lack of knowledge of human milk banking or concerns about the safety of DHM [28–30]. There is currently no human milk bank in Wenzhou, and understanding mothers’ acceptance of human milk banking is crucial to the establishment of HMBs in Wenzhou. Therefore, the aim of this study was to assess the prevalence and associated factors of women’s willingness to donate human milk and use DHM, which may help in developing targeted strategies to promote acceptance of human milk banking in Wenzhou.

## Methods

### Study design

A cross-sectional study using convenience sampling was conducted to explore prevalence and associated factors of willingness to donate human milk and use DHM among women living in Wenzhou in December 2020.

The study was carried out in three community health service centers in Wenzhou. Wenzhou, located in southeast China, is an important commercial city along the southeast coast with a resident population of 9.3 million and 98,000 births in 2019 [31]. These community health centers provide public health services and basic medical services and are government funded. They serve the community, families and residents with a focus on women, children, elderly individuals, chronically ill patients, disabled patients and poor residents, and they take the initiative to provide door-to-door services, health education, prevention, healthcare, rehabilitation, technical services for family planning, and the diagnosis and treatment of common and frequently occurring diseases [32]. Vaccination is one of their services. Children in China are required to receive the prescribed vaccinations on time from birth to the age of twelve. After birth, infants receive their first dose of hepatitis B and Bacillus Calmette–Guérin (BCG) vaccines in the hospital, while the rest of the vaccinations are performed at community health centers. Many vaccinations are required in the first year of an infant’s life, so many women bring their children under one year old to community health centers to be vaccinated.

### Inclusion and exclusion criteria

Women who brought their children to the vaccination departments of the three selected community health centers during the survey time were invited to participate. The inclusion criteria for the sample were as follows: age greater than or equal to 18 years, within one year after giving birth, singleton pregnancy, able to read and communicate, and informed and voluntary participation in the study. Women with mental disorders were excluded from this study.

### Sample size determination

The required sample size was determined using the single proportion population formula $n=\frac{Z_{a/2}^2P\left(1- pP\right)}{\delta^2}$ with the following assumptions: estimated prevalence of willingness to donate human milk 81.3% [30], critical value at 95% confidence interval (1.96), margin of error 6% and response invalidity 10%. The required sample size was calculated as 181. The estimated prevalence of willingness to use DHM should also be considered in the sample size determination since willingness to use DHM was another dependent variable in our study. The estimated prevalence of willingness to use DHM was 38.3% [30], and the remaining assumptions were the same as above; this yielded a sample size of 281. Taking into account the two dependent variables, the final required sample size for this study was determined to be 281. In the end, we surveyed 307 women. The numbers of participants surveyed at the Nanbaixiang Community Health Center, Nanpu Community Health Center and Shuangyu Community Health Center were 107, 100 and 100, respectively. Participants with response times shorter than 120 s were excluded.

### Data collection

#### Research instrument

After reviewing the literature, the researchers listed potential factors that might be associated with human milk donation or receiving DHM and then designed a survey instrument based on these influencing factors. The instrument for our study consists of five sections: 1) sociodemographic characteristics: age, education, employment (participants who were not in paid employment were defined as unemployed), monthly household income in the previous year and number of children in the household; 2) perinatal characteristics: parity, mode of delivery, the gestational age, birthweight, sex and age of the infant; 3) breastfeeding perception and awareness of human milk banking: the question “Is human milk nutritionally superior to infant formula?” was used to assess participants’ perception of breastfeeding, and two other questions asked participants if they had heard of human milk donation or HMB prior to this survey and where they obtained the information; 4) human milk banking knowledge: the Human Milk Banking Knowledge Questionnaire was adapted from previous studies [29, 33] and contained nine questions. Each question had three choices: “Yes”, “No” and “Do not know”, with one point for a correct answer and zero points for an incorrect or unknown answer; and 5) willingness to donate human milk and to receive DHM: participants were required to respond to two questions, “Are you willing to donate human milk?” and “Are you willing to receive donor human milk to feed your child?”. Participants were also asked to give reasons for their answers to the above willingness questions; participants who were willing to donate human milk were asked to answer the question “Are you willing to undergo a predonation blood test?”. In addition, all participants had to answer “Should human milk donors be rewarded with money?” and “What would you most like to know about human milk banking?”

A panel consisting of one senior nurse, two midwives and two NICU physicians was invited to review the initial survey instrument. Based on the recommendations of the panel members, overlapping questions were removed, and statements that were not in line with the conventions of expression were modified. The original five experts were then invited again to assess the content validity of the Human Milk Banking Knowledge Questionnaire, and the content validity index was 0.96. The survey was pilot tested with thirty women. The Cronbach’s alpha for the Human Milk Banking Knowledge Questionnaire was calculated to be 0.74 and the Spearman-Brown coefficient of split-half reliability was 0.71, which are considered satisfactory [34]. In the formal survey, Cronbach’s alpha and the Spearman-Brown coefficient of split-half reliability for the Human Milk Banking Knowledge Questionnaire were 0.83 and 0.76 based on the results of 305 participants.

#### Survey procedures

The survey instrument was entered into China’s largest online survey platform to generate a QR code for distribution of the survey. To ensure that all questions were answered, the online instrument was set up so that if a question was not answered, the survey could not be submitted. Prior to the survey, participants were briefed on the purpose and content of the study, and verbal consent was obtained. Participants could scan the QR code of the instrument with their own mobile phones and answer the online questions themselves. If participants had security concerns about the QR code provided by the researchers, they could use the researchers’ mobile phones to scan the QR code and complete the survey. If participants were unable to operate their own mobile phones, the researchers scanned the QR code with their own mobile phones, read the questions to the respondents one by one, and recorded the results of the participants’ verbal responses to the questions. Participants were not given any education about human milk banking by our researchers until they completed the survey.

### Data analysis

SPSS statistics 22 (IBM, Armonk, NY, USA) was used for data analysis. Numerical variables, including infant age and knowledge of human milk banking, did not follow a normal distribution and are represented by medians and quartiles (P25, P75). Categorical variables are described as cases (*n*) and percentages (%). In the univariable analysis, the Mann-Whitney U test and chi-square test were used for skewed continuous and categorical variables, respectively. Variables with *p* < 0.20 were included in the multivariable logistic regression model to identify independent predictors of dependent variables with the significance level set at *p* < 0.05 [35]. The variance inflation factor (VIF) was used to assess the potential presence of multicollinearity in the multivariable logistic regression. Variables with VIF < 5 were entered into the multivariable models and no collinearity was detected [36]. Variables with *p* < 0.05 were considered statistically significant in the multivariable logistic regression analysis. Unadjusted odds ratios (UORs) and adjusted odds ratios (AORs) were used to assess the association between independent variables and dependent variables.

## Results

### Characteristics of the sample

Of the 307 participants, 305 provided valid responses and two were excluded due to a short response time of 120 s. The sociodemographic, perinatal and breastfeeding characteristics of the participants are shown in Table 1.

**Table 1:** Sociodemographic, perinatal and breastfeeding Characteristics of Participants (*N* = 305)

Characteristics	n (%) or M [25,75%]
Age (year)	
≤ 25	60 (19.7)
26 ~ 30	120 (39.3)
31 ~ 35	100 (32.8)
> 35	25 (8.2)
Education	
Junior middle school or below	66 (21.6)
High school or technical secondary school	81 (26.6)
College	51 (16.7)
Bachelor degree	85 (27.9)
Master’s or above	22 (7.2)
Employment	
Unemployed	76 (24.9)
Employed	229 (75.1)
Monthly household income in the previous year, RMB	
≤ 5000	101 (33.1)
5001 ~ 10,000	115 (37.7)
> 10,000	89 (29.2)
Number of children in the household	
1	145 (47.5)
2	143 (46.9)
≥ 3	17 (5.6)
Parity	
Primiparous	154 (50.5)
Multiparous	151 (49.5)
Mode of delivery	
Vaginal	207 (67.9)
Cesarean	98 (32.1)
Gestational age of the infant (weeks)	
< 37	18 (5.9)
≥ 37	287 (94.1)
Birth weight of the infant (kg)	
≤ 2.5	14 (4.6)
2.5 ~ 4	245 (80.3)
> 4	46 (15.1)
Sex of the infant	
Female	148 (48.5)
Male	157 (51.5)
Age of the infant (months)	4 [2,8]
Is human milk nutritionally superior to formula?	
Yes	268 (87.9)
No	10 (3.3)
Do not know	27 (8.9)

Note: 1) M = median. 2) RMB is the Chinese currency and the exchange rate of EURO to RMB during the survey period was 1EURO = 8.0218RMB. 3) Mode of childbirth refers to the most recent delivery. 4) The infant mentioned refer to the child born to the respondent’s most recent birth

### Awareness and knowledge of human milk banking

Only 17.0% (52/305) of mothers had heard of human milk banking prior to the survey. Regarding information sources of human milk banking, hospital facilities (e.g., pamphlets, videos, bulletin boards) were most frequently mentioned, followed by the internet, friends or relatives, and healthcare professionals. Traditional public media, such as television, radio, magazines, and books, were rarely mentioned by participants (Table 2).

**Table 2:** Information sources of participants who had heard of human milk banking (*n* = 52)

Sources	Frequency (%)
Hospital facilities (pamphlets, video and bulletin boards, etc.)	17 (32.7)
Internet	14 (26.9)
Friends or relatives	8 (15.4)
Health care professionals	8 (15.4)
Television or radio	2 (3.8)
Magazines or books	1 (1.9)
Others	2 (3.8)

The median score for participants’ knowledge of human milk banking was 6, the 25th percentile was 3 and the 75th percentile was eight. Twenty-four participants scored 0, and twenty-nine scored nine. Table 3 shows the participants’ responses to the questions on knowledge of human milk banking. The item with the lowest percentage of correct answers was “Most nutrients in donor human milk are destroyed after pasteurization”, with 30.5% (93/305) of participants answering the question correctly. Respondents were most knowledgeable about donor health screening, with over three quarters answering this question correctly.

**Table 3:** Participants’ human milk banking knowledge responses (*N* = 305)

Questions	Yes n (%)	No n (%)	Do Not Know n (%)
1. Donor human milk is the best alternative for preterm infants when the mother’s own milk is insufficient or unavailable.	156 (51.1)	28 (9.2)	121 (39.7)
2. For preterm infants, donor human milk is nutritionally superior to formula.	216 (70.8)	25 (8.2)	64 (21.0)
3. Most nutrients of donor human milk are destroyed after pasteurization.	52 (17.0)	93 (30.5)	160 (52.2)
4. Feeding preterm infants with donor human milk facilitates their growth and recovery from disease.	202 (66.2)	13 (4.3)	90 (29.5)
5. Mothers who donate human milk are required to undergo a health screening.	239 (78.4)	2 (0.7)	64 (21.0)
6. Mothers who have excess human milk can donate it.	9 (3.0)	234 (76.7)	62 (20.3)
7. After pasteurization, donor human milk is stored frozen and is valid for 3 to 6 months.	171 (56.1)	17 (5.6)	117 (38.4)
8. Donor human milk should be stored in Human Milk Banks.	171 (56.1)	6 (2.0)	128 (42.0)
9. Donor human milk needs to be pasteurized before it can be given to infants.	166 (54.4)	28 (9.2)	111 (36.4)

Note: 1) Question 3 and 6 are reverse scoring questions, which scores zero points for “Yes” or “Do not know” and one point for “No”. 2) The number of correct answers was added to calculate a knowledge score for each participant

### Willingness to donate human milk and to accept DHM

Table 4 presents the willingness of participants to donate human milk and to accept DHM to feed their infants.

**Table 4:** Participants’ willingness to donate human milk and accept donor human milk

Variables	Frequency (%)
Are you willing to donate human milk? (*N* = 305)	
Yes	224 (73.4)
No	81 (26.6)
What is the main reason why you are willing to donate human milk? (*n* = 224)	
Helping other infants	159 (71)
There’s excess breast milk	52 (23.2)
Relating with religious charitable works	2 (0.9)
Increasing confidence in lactation	4 (1.8)
others	7 (3.1)
Are you willing to undergo a predonation blood test? (*n* = 224)	
Yes	194 (86.6)
No	8 (3.6)
Not sure	22 (9.8)
What is the main reason why you are unwilling to donate human milk? (*n* = 81)	
Lack of breast milk	47 (58.0)
Due to health problems, breast milk is not suitable for donation	7 (8.6)
Donor human milk may not be used effectively	2 (2.5)
Donation procedures are too complicated	11 (13.6)
Having no time	7 (8.6)
Knowing little of human milk banking	6 (7.4)
Others	1 (1.2)
Are you willing to receive donor human milk to feed your child? (*N* = 305)	
Yes	136 (44.6)
No	169 (55.4)
What is the main reason why you are willing to accept donor human milk for your infant? (*n* = 136)	
Human milk is nutritionally superior to formula milk	109 (80.1)
Pediatrician suggests using human milk	8 (5.9)
Infants nearby receiving donor human milk result in better health	11 (8.1)
others	8 (5.9)
What is the main reason why you are unwilling to accept donor human milk for your infant? (*n* = 169)	
Formula is as healthy for an infant as human milk	23 (13.6)
Concerns about inadequate health screening of human milk donors	73 (43.2)
Concerns about contamination of donor human milk in the process of expressing	36 (21.3)
Concerns about losing nutrients in pasteurization and preservation of donor human milk	13 (7.7)
others	24 (14.2)
Should human milk donor be rewarded with money? (*N* = 305)	
Yes	41 (13.4)
No	264 (86.6)
What would you most like to know about human milk banking? (128 = 305)	
Qualifications for human milk donor	140 (45.9)
Procedure of human milk donation	51 (16.7)
Who will benefit from human milk donation	33 (10.8)
Current status of human milk banks	61 (20.0)
others	20 (6.6)

A total of 73.4% (224/305) of respondents indicated that they were willing to donate human milk. The most common reason for willingness to donate human milk was to help other infants (71%, 159/224), while the top reason for reluctance to donate human milk was lack of human milk (58.0%, 47/81). Of those respondents who were willing to donate human milk, 86.6% (194/ 224) said they would be willing to undergo blood testing as part of a health screening prior to donation. When asked what they most wanted to know about human milk banking, 45.9% (140/305) of the participants responded with how to become a qualified donor, followed by the current status of HMBs (20%, 61/305), the human milk donation procedure (16.7%, 51/305), who uses DHM (10.8%, 33/305) and others (6.6%, 20/305). In addition, 86.6% (264/305) of respondents indicated that there was no need to reward human milk donors with money.

Fifty-five percent of mothers (55.4%, 169/305) were reluctant to use DHM to feed their own children. Of mothers who were willing to use DHM, 80.1% (109/136) perceived that DHM was nutritionally superior to formula. The most common reason (43.2%, 73/169) for reluctance to use DHM was concern about the lack of rigorous donor health screening.

### Factors associated with willingness to donate human milk

In the univariable analysis of factors associated with willingness to donate human milk, variables including education, employment, monthly household income in the previous year, number of children in the household, infant birthweight, age of the infant, awareness of human milk banking and knowledge of human milk banking (*p* < 0.20) were entered into the multivariable logistic regression model (Table 5).

**Table 5:** Univariable analysis of factors associated with willingness to donate human milk and accept donor human milk

	Willingness to donate human milk					Willingness to accept donor human milk				
	Yes *n* = 224	No *n* = 81	χ^2^ / Z	p	UOR(95%CI)	Yes *n* = 136	No *n* = 169	χ^2^/ Z	p	UOR(95%CI)
Age (year)			4.325	0.228				3.065	0.382	
≤25	42 (18.8)	18 (22.2)			1 (Reference)	22 (16.2)	38 (22.5)			1 (Reference)
26 ~ 30	93 (41.5)	27 (33.3)			1.476 (0.734,2.969)	56 (41.2)	64 (37.9)			1.511 (0.800,2.854)
31 ~ 35	68 (30.4)	32 (39.5)			0.911 (0.455,1.823)	44 (32.4)	56 (33.1)			1.357 (0.704,2.618)
> 35	21 (9.3)	4 (4.9)			2.250 (0.675,7.496)	14 (10.3)	11 (6.5)			2.198 (0.852,5.675)
Education			11.211	0.024				2.330	0.675	
Junior middle school or below	38 (17.0)	28 (34.6)			1 (Reference)	26 (19.1)	40 (23.7)			1 (Reference)
High school or technical secondary school	62 (27.7)	19 (23.5)			2.404 (1.184,4.885)	40 (29.4)	41 (24.3)			1.501 (0.777,2.899)
College	39 (17.4)	12 (14.8)			2.395 (1.065,5.386)	22 (16.2)	29 (17.2)			1.167 (0.556,2.452)
Bachelor degree	67 (29.9)	18 (22.2)			2.743 (1.344,5.598)	40 (29.4)	45 (26.6)			1.368 (0.712,2.625)
Master’s or above	18 (8.0)	4 (4.9)			3.316 (1.010,10.881)	8 (5.9)	14 (8.3)			0.879 (0.324,2.388)
Employment			3.436	0.064				1.199	0.289	
Unemployed	62 (27.7)	14 (17.3)			1 (Reference)	38 (27.9)	38 (22.5)			1 (Reference)
Employed	162 (72.3)	67 (82.7)			0.546 (0.286,1.042)	98 (72.1)	131 (77.5)			0.748 (0.445,1.259)
Monthly household income in the previous year, RMB			4.661	0.097				3.476	0.176	
≤5000	76 (33.9)	25 (30.9)			1.625 (0.867,3.045)	51 (37.5)	50 (29.6)			1.731 (0.969,3.093)
5001 ~ 10,000	90 (40.2)	25 (30.9)			1.924 (1.033, 3.583)	52 (38.2)	63 (37.3)			1.401 (0.796,2.466)
> 10,000	58 (25.9)	31 (38.3)			1 (Reference)	33 (24.3)	56 (33.1)			1 (Reference)
Number of children in the household			6.786	0.034				0.573	0.751	
1	111 (49.6)	34 (42.0)			1 (Reference)	62 (45.6)	83 (49.1)			1 (Reference)
2	105 (46.9)	38 (46.9)			0.846 (0.496,1.444)	67 (49.3)	76 (45.0)			1.180 (0.741,1.879)
≥3	8 (3.6)	9 (11.1)			0.272 (0.098,0.760)	7 (5.1)	10 (5.9)			0.937 (0.338,2.600)
Parity			1.614	0.243				0.148	0.730	
Primiparous	118 (52.7)	36 (44.4)			1 (Reference)	67 (49.3)	87 (51.5)			1 (Reference)
Multiparous	106 (47.3)	45 (55.6)			0.719 (0.431,1.198)	69 (50.7)	82 (48.5)			1.093 (0.696,1.716)
Mode of delivery			1.947	0.211				1.710	0.218	
Vaginal	147 (65.6)	60 (74.1)			1 (Reference)	87 (64.0)	120 (71.0)			1 (Reference)
Cesarean	77 (34.4)	21 (25.9)			1.497 (0.848,2.642)	49 (36.0)	49 (29.0)			1.379 (0.851,2.235)
Gestational age of the infant (weeks)			1.491	0.222				0.227	0.635	
< 37	11 (4.9)	7 (8.6)			1 (Reference)	9 (6.6)	9 (5.3)			1 (Reference)
≥ 37	213 (95.1)	74 (91.4)			1.832 (0.685,4.899)	127 (93.4)	160 (94.7)			0.794 (0.306,2.058)
Birth weight of the infant (kg)			3.967	0.138				0.520	0.771	
≤2.5	8 (3.6)	6 (7.4)			1 (Reference)	5 (3.7)	9 (5.3)			1 (Reference)
2.5 ~ 4	178 (79.5)	67 (82.7)			1.993 (0.666,5.957)	111 (81.6)	134 (79.3)			1.491 (0.486,4.578)
> 4	38 (17.0)	8 (9.9)			3.562 (0.967,13.131)	20 (14.7)	26 (15.4)			1.385 (0.401,4.780)
Gender of the infant			1.484	0.223				0.052	0.908	
Male	104 (46.4)	44 (54.3)			1 (Reference)	65 (47.8)	83 (49.1)			1 (Reference)
Female	120 (53.6)	37 (45.7)			1.372 (0.824,2.285)	71 (52.2)	86 (50.9)			1.054 (0.671,1.656)
Age of the infant (months)	5 (2,8)	3 (2,7)	1.669	0.095		5 (2,8)	4 (2,7)	0.887	0.375	
Breastfeeding perception			3.179	0.21				4.231	0.121	
Human milk is nutritionally superior to formula.	201 (89.7)	67 (82.7)			1 (Reference)	124 (91.2)	144 (85.2)			1 (Reference)
Formula is nutritionally superior to human milk.	7 (3.1)	3 (3.7)			0.778 (0.196,3.093)	5 (3.7)	5 (3.0)			1.161 (0.329,4.105)
I don’t know	16 (7.1)	11 (13.6)			0.485 (0.214,1.096)	7 (5.1)	20 (11.8)			0.406 (0.166,0.993)
Awareness of human milk banking.			2.750	0.097				13.094	0.000	
Yes	43 (19.2)	9 (11.1)			1 (Reference)	35 (25.7)	17 (10.1)			1 (Reference)
No	181 (80.8)	72 (88.9)			0.526 (0.244,1.135)	101 (74.3)	152 (89.9)			0.323 (0.172,0.607)
Knowledge of human milk banking	6 (4,8)	5 (2,7)	3.754	0.000		7 (5,8)	5 (3,7)	4.847	0.000	

Note: 1) RMB is the Chinese currency and the exchange rate of EURO to RMB during the survey period was 1EURO = 8.0218RMB. 2) Mode of childbirth refers to the most recent delivery. 3) The infant mentioned refer to the child born to the respondent’s most recent birth. 4) The number of correct answers was added to calculate a knowledge score for each participant. 5) UOR = unadjusted odds ratio, CI = confidence interval

Although parity exhibited *p* > 0.20 in the univariable analysis, we retained it in the multivariable analysis based on the existing literature [29, 37]. The Box-Tidwell test indicated that the relationship between the continuous independent variables (infant age and knowledge of human milk banking) and the dependent variable (willingness to donate human milk) was linear. After adjusting for potential confounders, employment and knowledge of human milk banking remained significant predictors of willingness to donate human milk at a *p* value cutoff of 0.05 (Table 6). According to the Hosmer-Lemeshow test, chi-square = 8.366, degrees of freedom (df) = 7, and *p* = 0.301. As the *p* value was greater than 0.05, the logistic model was adequate and fit the data. In addition, omnibus tests of model coefficients resulted in chi-square = 22.306, degrees of freedom (df) = 2, and *p* = 0.000, indicating that the forward stepwise (likelihood ratio) multivariable logistic model was very appropriate.

**Table 6:** Multivariable analysis of factors associated with willingness to donate human milk (*N* = 305)

Variables	AOR	95% CI	*P* value
Employment			
Employed	Reference		
Unemployed	2.297	1.172, 4.504	0.015
Knowledge of human milk banking	1.230	1.118, 1.354	0.000

Note: 1) The number of correct answers was added to calculate a knowledge score for each participant. 2) AOR = adjusted odds ratio

Respondents who were unemployed were more than twice as likely to be willing to donate human milk as those who were employed (AOR 2.30; 95% CI 1.17–4.50). Each point increase in the human milk banking knowledge score was associated with a 23.0% increase in mothers’ willingness to donate human milk.

### Factors associated with willingness to accept DHM

In the univariable analysis of factors associated with willingness to accept DHM, four variables, monthly household income in the previous year, perception of breastfeeding, awareness of human milk banking and knowledge of human milk banking (*p* < 0.20), were included in the multivariable logistic regression model (Table 5). The Box-Tidwell test results show a linear relationship between knowledge of human milk banking and willingness to use DHM. After adjusting for potential confounders, monthly household income in the previous year, awareness of human milk banking and knowledge of human milk banking were found to be significantly associated with willingness to accept DHM at a *p* value cutoff of 0.05 (Table 7). The results of the Hosmer-Lemeshow test (chi-square = 6.390, degrees of freedom (df) = 7, *p* = 0.495) and omnibus tests of model coefficients (chi-square = 35.520, degrees of freedom (df) = 4, *p* = 0. 000) suggested that forward stepwise (likelihood ratio) multivariable logistic regression was highly suitable.

**Table 7:** Multivariable analysis of factos associated with willingness to accept donor human milk (*N* = 305)

Variables	AOR	95% CI	*P* value
Monthly household income in the previous year			
> 10,000	Reference		0.049
5001 ~ 10,000	1.426	0.782,2.598	0.247
≤ 5000	2.175	1.166,4.057	0.015
Awareness of human milk banking.			
No	Reference		
Yes	2.408	1.242, 4.669	0.009
Knowledge of human milk banking	1.221	1.108, 1.345	0.000

Note: 1) The number of correct answers was added to calculate a knowledge score for each participant

Mothers with a monthly household income of up to ¥5000 in the previous year were twice (AOR 2.18; 95% CI 1.17–4.06) as likely to receive DHM to feed their children as those with a monthly household income of more than ¥10,000 in the previous year. The odds of willing to accept DHM among mothers who had heard of human milk banking was 2.4 times (AOR 2.41; 95% CI 1.24–4.67) higher than that of mothers who had not heard of human milk banking. Mothers’ willingness to use DHM increased by 22.1% with each point increase in the human milk banking knowledge score.

## Discussion

Awareness of human milk banking was low in our study, with only 17% of participants having heard of it prior to the survey. This finding is similar to those of studies conducted by Zhang (20%), Wang (28%) and Qin (16.7%) [29, 30, 33]. This may be related to the lack of publicity of human milk banking in the respondents’ cities at the time the survey was conducted. A study done in China by Tian et al. reported that 40.1% of the participants had heard of milk banks or donor milk; in their study, the recipients were provided with the survey link through WeChat, which is similar to Facebook, and those who were interested or concerned about DHM completed the questionnaires, which might result in a higher awareness rate [38]. In our study, the top source of human milk banking information among mothers was hospital facilities, in contrast to other studies that reported the internet as the primary information source of human milk banking [30, 38]. One possible explanation for this is that most online news outlets had not yet paid attention to human milk banking. Given the speed and reach of online media, full use should be made of authoritative media to promote human milk banking. In our study, many mothers were unsure of the nutritional value of pasteurized DHM, but participants in other studies had better knowledge of this item [28, 39]. This finding may be explained by the fact that our participants were unfamiliar with human milk banking.

In this study, 73.4% of mothers said they were willing to donate human milk. This is similar to the results of studies in other cities in China, which showed that 76.7 – 82.7% of participants were willing to donate human milk [29, 30, 40]. This result is also in line with a study conducted in Kenya, where 78% of respondents said they were willing to donate human milk [39]. However, a study conducted in semirural Turkey reported that only 19.1% of the women surveyed considered donating milk for banking; the main reason for reluctance to donate milk to the bank was religious concerns that using DHM restricts marriage potential between human milk donor children and recipient children [40]. The majority of the population in Wenzhou is not Muslim, and our study participants had no such religious concerns and therefore a higher willingness to donate human milk.

A total of 44.6% of participants in this study were willing to receive DHM to feed their children, which is similar to the prevalence of willingness to use DHM reported in other studies conducted in China [29, 30]. The reluctance to accept DHM might be because participants in our study were mothers of healthy infants and had no real need for DHM; in addition, they were concerned about inadequate screening of human milk donors, which is consistent with safety concerns reported in other studies [29, 30, 39, 41–43]. The results of a study in Ethiopia showed that participants’ willingness to use DHM was particularly low, with only 15.2% of participants willing to use DHM, which might be related to the lower awareness (10%) of human milk banking [42].

In our study, employment and knowledge of human milk banking were two independent predictors of willingness to donate human milk. Concerning employment, our findings indicate that unemployed mothers were more willing to donate human milk than mothers who were employed. This was supported by the findings of Jang’s study, which reported that 62.3% of the donors were housewives in the analysis of 463 human milk donors at the human bank in Kyung Hee University Hospital [38]. This might be related to the fact that unemployed mothers in our study had more free time and were able to participate in predonation health screening and human milk collection at an HMB. Our study also showed that willingness to donate human milk increased as mothers had more correct knowledge about human milk banking. This is consistent with the findings from a study in southeast Nigeria [44], which indicated that knowledge of DHM was predictive of participants who would be potential human milk donors. The reason might be that proper understanding of a concept could facilitate people accepting it [44]. In our study, parity was not significantly associated with women’s willingness to donate human milk. However, the results of other studies [29, 45] indicated that multiparous mothers had a positive attitude toward human milk donation. A possible explanation for the difference might be that new mothers in our study, although inexperienced, learned about the benefits of human milk through breastfeeding promotion programs provided by the government and hospitals, made their own efforts to breastfeed, and supported other mothers in feeding their infants with human milk.

We also found in this study that monthly household income in the previous year, awareness of human milk banking and knowledge of human milk banking were significantly associated with participants’ willingness to use DHM to feed their infants. Our findings indicated that the willingness to use DHM decreased with monthly household income in the previous year. In contrast, a study done in Vietnam showed that families with a higher income were more likely to use pasteurized DHM to feed their healthy newborns [45]. The difference might be explained by the fact that the mothers in our study were unfamiliar with human milk banks and were unsure of the safety of DHM, whereas in good economic circumstances, they would rather afford expensive but relatively safe infant food, such as formula, to feed their infants. In this study, mothers who had heard about human milk banking and were more knowledgeable about human milk banking were more likely to use DHM. This finding is similar to the results of a study performed in Ethiopia and Nigeria [42, 44]. The possible reason for this might be that as awareness and knowledge of human milk banking increased, mothers in our study were more confident in the quality and safety of DHM, and therefore, their willingness to use human milk increased.

### Strengths and limitations

The main strength of this study is that we explored both the factors that influence women’s willingness to donate human milk and the factors associated with their willingness to use DHM. There are some limitations to our study. First, the survey instrument in our study was closed-ended, which limited participants’ opportunity to express their opinions that were different from the options we provided. Furthermore, some factors that may influence the acceptance of human milk banking were not included, such as family support for human milk banking and religious beliefs. Second, the study was conducted on a convenience sample of mothers from one city and the number of mothers who refused to participate in the survey was not recorded. Therefore, the results of our study are not applicable to postpartum women in other cities in China and stronger evidence is still needed to confirm our findings. A multi-center study with a large sample size could increase the generalizability of the study results.

## Conclusions

In our community sample of mothers in China, awareness of human milk banks among women in the first year postpartum was low, and the main source of information was hospital institutions. More mothers were willing to donate human milk than use DHM to feed their children. Employment and knowledge of human milk banking were independently associated with women’s willingness to donate human milk in our study. Monthly household income in the previous year, awareness of human milk banking and knowledge of human milk banking were significant predictors of mothers’ willingness to accept DHM. Therefore, we suggest that the government, community and hospitals provide mothers with detailed information on human milk banking through various channels to help improve their knowledge of human milk banking and thus increase the acceptance of human milk banking. In addition, there is also a need for qualitative research on this topic in the NICU.

## Data Availability

The datasets analyzed during the current study are available from the corresponding author on reasonable request.

## Abbreviations

AOR: Adjusted odds ratio
CI: Confidence interval
DHM: Donor human milk
HMB: Human milk bank
NICU: Neonatal intensive care unit
UOR: Unadjusted odds ratio
